# Diagnostic Performance of Visionix VX120+ Platform for Dry Eye Screening

**DOI:** 10.3390/diagnostics14202276

**Published:** 2024-10-12

**Authors:** Elena Martínez-Plaza, Laura Barberán-Bernardos, Ainhoa Molina-Martín, David P. Piñero

**Affiliations:** 1 Group of Optics and Visual Perception, Department of Optics, Pharmacology and Anatomy, University of Alicante, 03690 Alicante, Spain; emartinezp@ioba.med.uva.es (E.M.-P.); laura.barberan@ua.es (L.B.-B.); ainhoa.molina@ua.es (A.M.-M.); 2 University of Valladolid, 47001 Valladolid, Spain; 3 Department of Ophthalmology, Vithas Medimar International Hospital, 03016 Alicante, Spain

**Keywords:** tear meniscus height, TMH, tear break-up time, NIBUT, accuracy, Visionix, dry eye, screening

## Abstract

Objective: To evaluate the accuracy of diagnosing dry eye disease (DED) by using the Visionix VX120+, to establish reference values for tear meniscus height (TMH) and non-invasive break-up time (NIBUT), and to compare the NIBUT measurements with the fluorescein tear break-up time (FBUT), Methods: fifty-eight subjects (34 dry eye and 24 control) were enrolled. The TMH, first NIBUT, and NIBUT50% were evaluated with the Visionix VX120+, and the FBUT was measured with a slit-lamp. The Receiver Operating Characteristic (ROC) curve was used to evaluate the diagnostic performance, and the Bland–Altman method was performed to analyze the agreement. Results: The areas under the curve were 0.62, 0.60, and 0.70 for the TMH, first NIBUT, and NIBUT50%, respectively. The optimal cut-off values (sensitivity, specificity) were 0.29 (0.62, 0.67), 5.05 (0.85, 0.46), and 7.35 (0.65, 0.79) for the TMH, first NIBUT, and NIBUT50%, respectively. The mean differences (lower, upper limits of agreement) were −1.10 (−8.78, 6.58) and 1.55 (−5.68, 8.78) for the first NIBUT vs. FBUT and the NIBUT50% vs. FBUT, respectively. Conclusions: In conclusion, the NIBUT50% can be a useful tool for dry eye screening, with acceptable values of sensitivity and specificity. First, the NIBUT and NIBUT50% should not be used interchangeably with the FBUT.

## 1. Introduction

Dry eye disease (DED) is a multifactorial condition of the ocular surface and is characterized by the loss of homeostasis of the tear film and the presence of ocular symptoms [1]. The prevalence of DED varies from 5 to 50% [2], depending on the population, as the prevalence is higher with age and among women [3]. 

Tear stability and volume can be affected in patients with DED [4]. With every blink, the tear spreads throughout the ocular surface, creating the tear film, and approximately 75–90% of the tear volume is held in the tear meniscus [5]. Therefore, measuring the tear meniscus height (TMH) can be a valuable sign in the diagnosis of DED [6,7,8]. In regard to the tear stability evaluation, the fluorescein tear film break-up time (FBUT) is still one of the most common tests used in the diagnosis of DED due to its accessibility and affordability to eye care professionals [9,10,11]. This test detects the time it takes for the tear film to evaporate after blinking by instilling fluorescein. However, this is an invasive and subjective measurement dependent on the observer. Moreover, the instillation of fluorescein can alter the tear film stability or produce reflex tearing [12,13], and the volume used can alter the FBUT measurements [14,15]. Therefore, several devices to objectively assess the non-invasive break-up time (NIBUT), such as dry eye modules of corneal topography systems, have been developed in recent years [16,17,18,19]. One of these instruments is the multi-diagnostic platform Visionix VX120+ (Visionix-Luneau Technologies, Chartres, France), which features a Dry Eye module that allows an objective measurement of break-up time. In particular, this device provides the first NIBUT (the time elapsed from the initial reflection until the first sign of Placido disk distortion) and NIBUT50% (the time elapsed from the initial reflection until half of the projected subareas become distorted), and a subjective measurement of the TMH. A previous study showed acceptable intrasession repeatability of the first NIBUT and TMH, as well as good interobserver reproducibility for the TMH [20]. Despite the results obtained for the NIBUT and TMH, which are not interchangeable to those provided by the system Medmont E300 (Medmont International Pty Ltd., Melbourne, Australia) [21], no studies have determined the interchangeability with the traditional method, the FBUT, which is measured with a slit-lamp biomicroscopy.

The objective of this study is to evaluate the accuracy and establish reference values for diagnosing DED, using the Dry Eye screening module of Visionix VX120+, which is based on the TMH and NIBUT measurements. A secondary objective is to compare the NIBUT measurements obtained from the Visionix VX120+ platform with those obtained with the standard procedure (FBUT) in subjects with and without DED.

## 2. Materials and Methods

This cross-sectional study was developed in the Optometry Clinic of the University of Alicante (Alicante, Spain), and it followed the tenets of the Declaration of Helsinki. The study was explained in detail to participants, who then signed a written informed consent. This study was approved by the Ethics Committee of the University of Alicante.

### 2.1. Sample

The general inclusion criteria were a consecutive sample of adult subjects with a corrected distance visual acuity lower or equal to 0.10 logarithm of the minimum angle of resolution (logMAR). Additionally, the sample was divided into two groups: the DED group and the control group. For the clinical diagnosis of DED, subjects within this group needed to have an ocular surface disease index (OSDI) score equal or greater than 13 and at least 1 eye that met at least one of the following criteria [9]: (i) Schirmer I test without anesthesia lower than 10 mm at 5 min; (ii) FBUT lower than 10 s; (iii) Grade 1 or higher in the Oxford scale of corneal and conjunctival staining. Subjects that did not meet these criteria in any eye constituted the control group.

All subjects that met any of the exclusion criteria were excluded from participation in the study. The general exclusion criteria were as follows: (i) No history of ocular pathology different from DED; (ii) No history of ocular surgery in the previous 6 months; (iii) presence of corneal alterations that could influence study results; (iv) Chronic medical treatment with drugs such as local/systemic anti-inflammatory drugs, local/systemic antibiotic drugs or local/systemic antihistaminic drugs.

### 2.2. Clinical Measurements

A visual examination, including subjective refraction and corrected distance visual acuity, was performed. In addition, the OSDI questionnaire was administered to evaluate the symptomatology associated with DED experienced by the participant over the past week, obtaining a score ranging from 0 to 100 points [22].

The Visionix VX120+ multi-diagnostic device consists of a Scheimpflug camera, a Hartmann-Shack sensor, Placido disks, and an air-puff system. In addition, the VX120+ incorporates a Dry Eye module, which allows the evaluation of the first NIBUT, the NIBUT50% (the time when half of the subareas of projection present distortion), and the TMH (manually measured using the caliper tool from a high-resolution photography).

Fluorescein dye (Bio Fluoro, Biotech Europe Meditech Inc Limited, Gallowstown, Ireland) was instilled in order to determine FBUT and ocular surface staining, observing with a slit-lamp biomicroscope. To determine FBUT, three consecutive measurements were performed and the mean value was calculated. Corneal, nasal conjunctival, and temporal conjunctival staining were graded using the Oxford scale, ranging from grade 0 to grade 5 [23]. The Schirmer I test (Bio Fluoro Biotech, Biotech Europe Meditech Inc Limited, Ireland) was performed without anesthesia for 5 min.

### 2.3. Dry Eye Module VX120+

The Dry Eye module of the VX120+ system performs a series of consecutive automated measures following this sequence in each eye: automated measurement of the NIBUT, high-definition photography of the anterior eye for analyzing the TMH, and finally, the acquisition of manual photographs by the examiner with the camera which is used for the grading of the severity of anterior segment alterations using a short version of the Efron scale [20].

The automated measurement of the NIBUT is obtained from the analysis of the projection of Placido disks used for the corneal topography exam on the anterior cornea surface. Specifically, after two consecutive blinks, the measurement procedure is initiated while subjects keep their eyelids open for the maximum possible period. The measurement procedure was automatically stopped after any blink during this procedure. A digital analysis of the images recorded is then performed, with a subdivision of the corneal area on which the projection was made in small subareas for a more detailed analysis. From this analysis, we detected the time point when the first distortion of the rings projected is produced due to a tear film break-up, which is called the first NIBUT. Likewise, the time when half of the subareas of projection analyzed is showing distortion is also calculated, which is called the NIBUT50%. It should be noted that if the subject blinks before the presence of distortions in the projection of the Placido disks due to the tear film break-up, this blink is considered as the break-up point, and automatically, this time point will be considered as the NIBUT and NIBUT50%. In the current study, the measurement was repeated in such cases to ensure its reliability. According to a previous study from our research group, a within-subject standard deviation of repeated measures of 0.9 and 1.4 s was found for NIBUT and NIBUT50%, respectively [20].

Once the system automatically provides the measurement of the NIBUT, the examiner can also manually obtain a measurement of the TMH. This is achieved from the high-resolution image obtained with the camera. Specifically, the examiner can define the limits of the meniscus with a digital caliper provided by the software of the system. Despite being the procedure manual, a within-subject standard deviation of repeated measures of 0.05 mm was found in our previous study evaluating the reliability of this system [20]. Likewise, in this previous research, no significant differences were found between two different examiners in the TMH measure, with an inter-examiner range of agreement of 0.12 mm [20].

Finally, the system offers the possibility of obtaining different anterior segment images in different gaze positions. These images can be used afterward to perform with a shortened version of the Efron scale, a subjective grading of several types of signs, including conjunctival redness, limbal redness, blepharitis, and meibomian gland dysfunction.

### 2.4. Statistical Analysis

The statistical analysis was performed using SPSS statistical package version 28.0.0 (IBM SPSS Inc., Chicago, IL, U.S.). Only one eye from each subject participating in this study was considered for analysis. For subjects who met the diagnosis criteria in only one eye, that eye was selected for analysis, and for subjects whose both eyes met the criteria, the analyzed eye was randomly selected.

Descriptive values were mean and standard deviation (SD) or median and interquartile (IQ) range for numeric and ordinal variables, respectively. Differences between the DED and control groups were analyzed using the independent Student-T test if the normality assumption was accomplished with the Shapiro-Wilk test; otherwise, the Mann-Whitney U test was used. 

The Receiver Operating Characteristic (ROC) curve [24] was used to evaluate the diagnostic performance of the first NIBUT, NIBUT50%, and TMH. The area under the ROC curve (AUC) and its 95% confidence interval (CI) were calculated. The optimal cut-off value, sensitivity, and specificity for each parameter were determined based on Youden’s index.

The agreement between the measurement methods (e.g., the tear break-up time with Visionix vs. slit-lamp biomicroscopy) was evaluated using the Bland–Altman approach [25]. The 95% limits of agreement (LoA) were determined as the mean difference of ±1.96 SD. Differences between measurement methods were analyzed using the paired Student-T test or the Wilcoxon test, depending on the normality assumption. *p*-values equal or lower than 0.05 were considered as statistically significant.

## 3. Results

A total of 58 eyes (30 right and 28 left eyes) of 58 subjects (38 females and 20 males) with a mean age of 48.8 ± 13.4 years were evaluated. The DED group included 34 eyes (16 right and 18 left eyes) of 34 subjects (24 females and 10 males) with a mean age of 52.2 ± 12.4 years, and the control group included 24 eyes (14 right and 10 left eyes) of 24 subjects (14 females and 10 males) with a mean age of 44.0 ± 13.5 years. No significant differences were found between groups for sex (*p* = 0.33), but significant differences were observed for age (*p* = 0.021). Table 1 shows descriptive data from both groups.

### 3.1. Diagnostic Accuracy of Visionix VX120+

**Figure 1:**
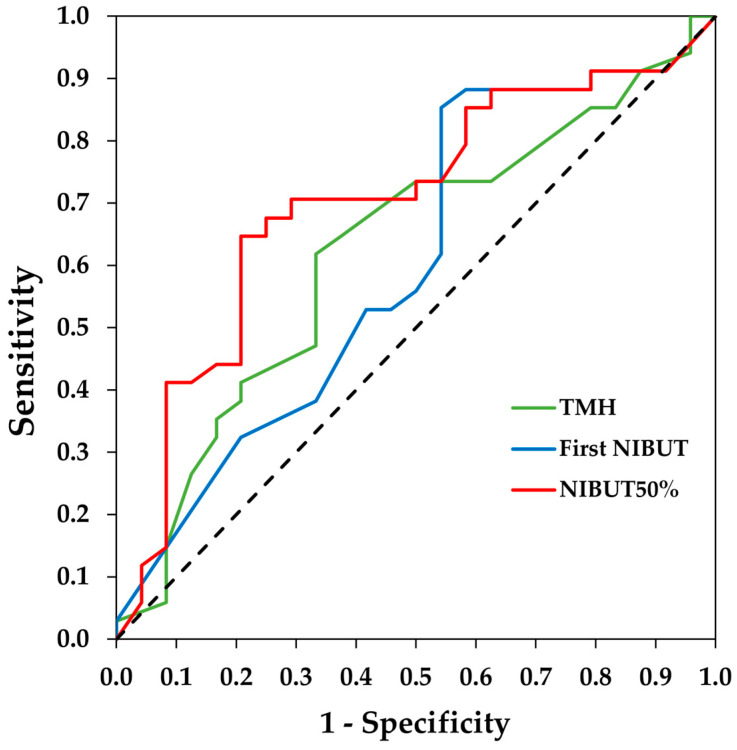
Receiver operating characteristic (ROC) curves of tear meniscus height (TMH), first non-invasive break-up time (NIBUT), and NIBUT50% measured with the Visionix VX120+ for the diagnosis of dry eye. The dotted line denotes the ROC curve if the classification is randomly estimated.

The area under the curve (AUC) and the 95% CI of the ROC curves were 0.62 (0.48/0.77) for the TMH, 0.60 (0.45/0.75) for the first NIBUT and 0.70 (0.56/0.84) for the NIBUT50%. The ROC curves are shown in Figure 1. The optimal cut-off values and their sensitivity and specificity are provided in Table 2.

### 3.2. Agreement between Visionix and Fluorescein Break-Up Time Measurements

**Figure 2:**
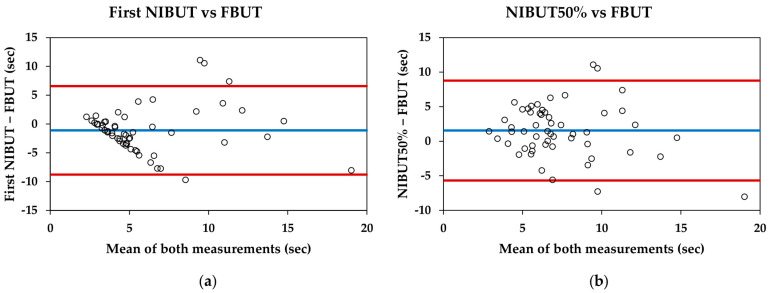
Bland–Altman plots comparing the Visionix VX120+ and fluorescein break-up time measurements: (**a**) First non-invasive break-up time (NIBUT) measured with VX120+ and fluorescein break-up time (FBUT) measured with slit-lamp; (**b**) NIBUT50% measured with VX120+ and FBUT measured with slit-lamp. Blue lines represent the mean difference between measurements, whereas red lines represent the 95% limits of agreement.

For the entire sample, the first NIBUT was significantly lower than the FBUT (5.34 ± 3.95 s and 6.44 ± 3.82 s, respectively; *p* = 0.007), and the NIBUT50% was significantly higher than the FBUT (7.99 ± 3.22 s and 6.44 ± 3.82 s, respectively; *p* = 0.002). The Bland–Altman data and plots are shown in Table 3 and Figure 2, respectively.

## 4. Discussion

In recent years, the development of objective instruments for dry eye detection has been increasing. Most of them are based on obtaining objective measures characterizing the tear film, such as the NIBUT and TMH [4]. The idea is to provide a fast and easy-to-perform diagnostic approach to dry eye that could be easily implemented in clinical practice. As the NIBUT can be measured through observations of Placido disk images that are reflected on the anterior ocular surface, currently marketed corneal topography systems have implemented this type of digital analysis [9,20,21]. Likewise, the incorporation of other examination modules in topography systems to create multi-diagnostic platforms has gained a great advance and opportunity for developing systems, allowing the detection of conditions in which the diagnosis can only be performed by comparing the results of different tests. The Visionix VX120+ enables a reliable evaluation of the first NIBUT, NIBUT50%, and TMH through its Dry-Eye module [20]. In this study, the diagnostic capacity of these parameters for DED detection has been investigated, establishing their optimal cut-off values. In addition, the interchangeability of tear film break-up time with the traditional method (FBUT with slit-lamp biomicroscopy) has been analyzed.

In the present study, case (DED subjects) and control (healthy subjects) groups were recruited, exhibiting the cases a higher mean age and a greater proportion of females; the difference in age was statistically significant. These characteristics align with the well-established demographic risk factors for DED [2]. Some observed ocular surface characteristics were different between the DED and the control group. Specifically, DED participants showed higher symptomatology, a shorter tear break-up time, and greater epithelial damage compared to the controls. Although symptoms and signs are known not to be well-correlated, these findings are consistent with previous literature [26,27].

The TMH demonstrated weak diagnostic accuracy for DED (AUC = 0.62) with an optimal cut-off value of 0.29 mm and sensitivity and specificity values of 62% and 67%, respectively. Vigo et al. [28] also assessed TMH using high-resolution photography, finding a similar AUC (0.61) with a slightly lower cut-off point (0.23 mm), and with similar specificity (63%) and sensitivity (57%). Singh et al. [7], using optical coherence tomography (OCT), identified a cut-off point of 0.21 mm for the TMH. Nonetheless, they reported higher diagnostic accuracy for detecting DED, which could be related to the greater precision of OCT, allowing a better identification of the upper and lower limits of the tear meniscus, compared to a high-resolution photography. Consequently, the TMH appears to be a valuable diagnostic parameter for DED, although its accuracy is highly dependent on the measurement instrument used.

Regarding the break-up time diagnostic accuracy of Visionix VX120+ for DED, we obtained a weak AUC (0.60) for the first NIBUT and a moderate AUC (0.70) for the NIBUT50%. Therefore, the data from this study suggest that the NIBUT50% has a better diagnostic capability for dry eye than the first NIBUT and TMH. Additionally, the sensitivity (65%) and specificity (79%) for the NIBUT50% are more balanced than for the first NIBUT. The better performance of the NIBUT50% may be because dry eye patients not only exhibit an earlier initial tear break-up (first NIBUT) but also continue to experience faster subsequent break-up up to at least half of the surface break up (NIBUT50%). The AUC values for the NIBUT50% found are higher than those reported for the FBUT (AUC = 0.60) [29] but lower than those for the NIBUT measured with other devices using Placido disks (0.83 or higher) [28,30,31,32]. Regarding the cut-off point for the NIBUT50%, our results indicate that the Visionix VX120+ has the most optimal cut-off point at 7.35 s. In other studies using different instruments, the cut-off values vary widely [28,30,31,32]; however, this could be attributed to the methodological differences in the projection of Placido disks and the algorithms used by each instrument to calculate tear break-up time.

Comparing the break-up time of Visionix VX120+ with the traditional measure (FBUT), the VX120+ provides lower mean values for the first NIBUT and higher mean values for the NIBUT50%. Therefore, neither of these parameters are interchangeable with the traditional method. Bland–Altman plots reveal that for the first NIBUT (Figure 2a), the pattern shows that the difference between measurements increases as the tear break-up time extends. This pattern has been previously described in studies involving healthy subjects and contact lens users [33,34]. However, the pattern observed between the NIBUT50% and FBUT is more consistent, with an approximate difference of one and a half seconds (Figure 2b). This could indicate that while the measurement of the first NIBUT may be influenced by other factors, the NIBUT50% could be more representative of the traditional measurement used in clinical practice.

One limitation of this study is the difference in mean age between the DED and control groups. However, this difference reflects everyday clinical practice, as age is a well-established risk factor for DED [3]. Another limitation may be not considering the DED subclassification (evaporative or acuodeficient types) for assessing the diagnostic accuracy of Visionix VX120+. However, the objective of this type of system is not to provide an accurate diagnosis of dry eye as this would require a complete ophthalmological examination. The objective of all these platforms is to make an efficient screening of the condition and recommend a more exhaustive analysis in those cases that are necessary. Future studies with larger sample sizes could be conducted to confirm if, besides the screening function of these systems, a differentiation in terms of dry eye subtype could be performed. Another drawback is that the measurements were conducted during the day, which may have introduced a slight variability to the study parameters. However, these conditions mimic those of the clinical practice, where patients attend throughout the day. Finally, the reference measurement to compare the TMH values obtained using the Visionix VX120+ with the most reliable method is lacking [35]. Unfortunately, we did not have access to an OCT to take that measurement during the study. In future studies, this comparative analysis should be performed.

## 5. Conclusions

The NIBUT50% provided by the Visionix VX120+ exhibits better diagnostic capability for dry eye diagnosis than the first NIBUT and TMH. Additionally, the acceptable values of sensitivity and specificity make this parameter a useful tool for dry eye screening. Finally, the tear break-up time measured with the Visionix VX120+ (i.e., the first NIBUT and NIBUT50%) could not be used interchangeably with the traditional fluorescein tear break-up time. Therefore, the cut-off values obtained in the current series cannot be considered as applicable to other systems, which measure the same parameters, as different detection algorithms could be used.

## Figures and Tables

**Table 1:** Descriptive data and comparison between the DED and control groups.

Parameters	DED Group	Control Group	*p*-Value
Spherical equivalent (D)	−1.18 ± 2.51	−0.25 ± 2.92	0.12
Visual acuity (logMAR)	−0.05 ± 0.09	−0.03 ± 0.11	0.81
OSDI questionnaire	37.91 ± 18.97	8.37 ± 9.66	<0.001
Tear meniscus height (mm)	0.27 ± 0.08	0.30 ± 0.08	0.13
First NIBUT (s)	4.62 ± 3.53	6.36 ± 4.34	0.19
NIBUT50% (s)	7.20 ± 3.12	9.11 ± 3.08	0.010
Fluorescein tear break-up time (s)	5.13 ± 1.95	8.29 ± 4.97	0.012
Corneal staining (Oxford scale)	0.00 [0.00/1.00]	0.00 [0.00/0.00]	0.051
Nasal conjunctival staining (Oxford scale)	0.00 [0.00/0.00]	0.00 [0.00/0.00]	0.60
Temporal conjunctival staining (Oxford scale)	0.00 [0.00/1.00]	0.00 [0.00/0.00]	0.035
Schirmer test (mm)	16.17 ± 11.26	18.33 ± 9.42	0.25

D: diopters; DED: dry eye disease; logMAR: logarithm of the minimum angle of resolution; NIBUT: non-invasive break-up time; OSDI: ocular surface disease index. Data are presented as mean ± standard deviation or median [percentile 25 to 75]. *p*-value indicates the comparison between groups.

**Table 2:** Optimal cut-off values, sensitivity and specificity for the dry eye parameters evaluated with the Visionix VX120+ platform.

Parameters	Optimal Cut-Off	Sensitivity	Specificity
Tear meniscus height (mm)	0.29	0.62	0.67
First NIBUT (s)	5.05	0.85	0.46
NIBUT50% (s)	7.35	0.65	0.79

NIBUT: non-invasive break-up time.

**Table 3:** Bland–Altman data summary for break-up time measurements.

Comparison	Mean Difference (95% CI)	Lower LoA (95% CI)	Upper LoA (95% CI)
First NIBUT vs. FBUT (s)	−1.10 (−2.11/−0.09)	−8.78 (−10.51/−7.06)	6.58 (4.86/8.31)
NIBUT50% vs. FBUT (s)	1.55 (0.60/2.50)	−5.68 (−7.30/−4.06)	8.78 (7.15/10.40)

CI: confidence interval; FBUT: fluorescein break-up time; LoA: limit of agreement; NIBUT: non-invasive break-up time.

## Data Availability

Data are available on request from the authors.
